# Patient-reported outcome measures for cancer caregivers: a systematic review

**DOI:** 10.1007/s11136-016-1239-0

**Published:** 2016-02-12

**Authors:** Valerie Shilling, Lucy Matthews, Valerie Jenkins, Lesley Fallowfield

**Affiliations:** Sussex Health Outcomes Research and Education in Cancer, Brighton and Sussex Medical School, University of Sussex, Falmer, Brighton, BN1 9QG UK

**Keywords:** Caregivers, Cancer, Outcome measures, Impact, Burden, Psychometric performance

## Abstract

**Purpose:**

Informal caregivers provide invaluable help and support to people with cancer. As treatments extend survival and the potential burdens on carers increase, there is a need to assess the impact of the role. This systematic review identified instruments that measure the impact of caregiving, evaluated their psychometric performance specifically in cancer and appraised the content.

**Methods:**

A two-stage search strategy was employed to: (1) identify instruments that measure the impact of caregiving, and (2) run individual searches on each measure to identify publications evaluating psychometric performance in the target population. Searches were conducted in MEDLINE, EMBASE, CINAHL and PsycINFO and restricted to English for instrument used and article language. Psychometric performance was evaluated for content and construct validity, internal consistency, test–retest reliability, precision, responsiveness and acceptability. Individual scale items were extracted and systematically categorised into conceptual domains.

**Results:**

Ten papers were included reporting on the psychometric properties of eight measures. Although construct validity and internal consistency were most frequently evaluated, no study comprehensively evaluated all relevant properties. Few studies met our inclusion criteria so it was not possible to consider the psychometric performance of the measures across a group of studies. Content analysis resulted in 16 domains with 5 overarching themes: lifestyle disruption; well-being; health of the caregiver; managing the situation and relationships.

**Conclusions:**

Few measures of caregiver impact have been subject to psychometric evaluation in cancer caregivers. Those that have do not capture well changes in roles and responsibilities within the family and career, indicating the need for a new instrument.

**Electronic supplementary material:**

The online version of this article (doi:10.1007/s11136-016-1239-0) contains supplementary material, which is available to authorized users.

## Background

Informal caregivers, whether they are spouse, family member or friend, often provide a significant amount of help and support for people with cancer. Informal caregiving is pivotal to the overall outcome of a patient’s treatment, and thus, maintaining the health and satisfaction of caregivers is essential to maximise the well-being of both parties [1, 2]. Caregiving can undoubtedly place a strain on the caregiver [3], but the role can also provide a source of happiness and boost self-efficacy and a sense of worth [4].

An increasing number of patients are living a longer life with cancer. As such, there is a growing recognition that broader aspects of their lives and those of the family are affected across the disease trajectory [5]. Informal caregivers could be viewed as “second-order patients in their own right” [6]; consequently, a well-validated measure to assess the impact of disease and treatment on their lives and overall well-being is crucial.

There is a raft of measures designed to assess caregiver impact so the choice for researchers may be unclear. The instruments currently used focus on three areas: caregiver burden, caregiver need and quality of life. Some measures are not well validated, and many have been developed for use with caregivers in very different circumstances, for example the elderly with cognitive impairment [7]. In order to better inform researchers on the content and evaluation of commonly used instruments, we identified and evaluated the psychometric performance of measures used in the cancer caregiver population and appraised their content, what is and what is not captured, with particular regard to broader areas of life experience such as the impact on career and family.

## Methods

The review involved a two-stage search: (1) to identify generic and cancer-specific self-report instruments used to measure the impact of caregiving on informal caregivers, and (2) to identify evidence about psychometric properties and performance of these instruments in the specific context of cancer.

### Search stage 1: Identifying candidate instruments

A combination of controlled syntax (MeSH) and free-text terms were used. Four groups of terms were generated: (1) generic names for measures; (2) impact on caregiver; (3) describing the population; and (4) psychometric performance. OvidSP was used for MEDLINE [MEDLINE(R) In-Process & Other Non-Indexed Citations and MEDLINE(R) 1946 to Present] and EMBASE (1947–current) searches. EBSCO*host* was used for CINAHL (1937–present) and PsycINFO (from 1800s to present) searches. Terms were modified as appropriate for each database and limited to English language only. Searches were run on 20 November 2014 (see “Appendix 1” for the search strategy used for MEDLINE, adapted for other databases).

### Study selection criteria

Inclusion criteria for stage 1 were self-report instruments of the impact of caring for patients with cancer or any other condition on the caregiver. Searches were not limited by study design or date, but were restricted to articles in the English language.

Instruments were excluded if they were developed: (1) to address a broad population not specifically for caregivers; (2) to focus on caregivers of children or children who are themselves caregivers; (3) to be administered only by an interviewer or clinician; (4) to measure unmet needs or objective aspects of caregiving, e.g. the amount of time or nature of tasks fulfilled; (5) to evaluate caregivers’ assessment or beliefs about their caregiving skills or performance; (6) for use in a non-English-speaking population and for which an English version was not available; (7) for use by patients rather than caregivers (e.g. patient estimates of the impact of their illness on the caregiver); and/or (8) to measure caregiver bereavement.

**Fig. 1:**
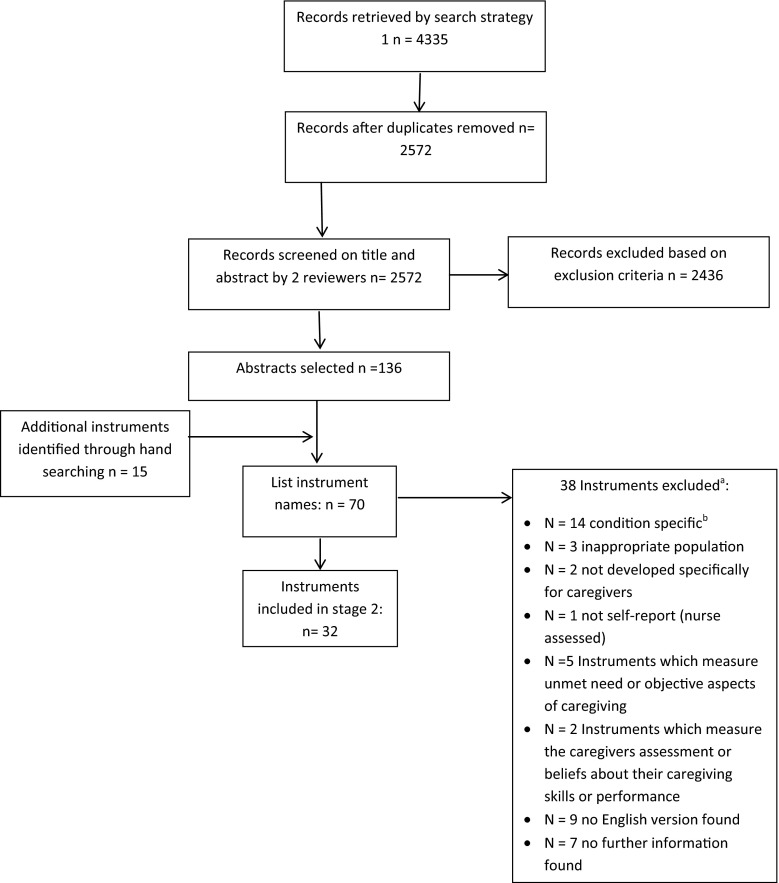
Flow chart showing identification and selection of potentially eligible instruments. ^a^ Five measures were excluded for more than one reason. ^b^ Where measures were developed for a specific group such as the frail elderly, stroke, dementia but could be used or adapted, we checked for its use in the cancer caregiver population (in the English language) before excluding it on this criterion

Titles and abstracts were screened independently by two reviewers (VS/LM) for names of instruments that met the inclusion criteria, resulting in a list of eligible candidate instruments (Fig. 1).

### Search stage 2: Identifying evidence of the psychometric properties of candidate instruments in the cancer caregiver population

Separate searches were conducted for each of the candidate instruments for studies designed to evaluate their psychometric performance in caregivers of cancer patients. Search terms are grouped as follows: (1) names and acronyms of the candidate instruments identified in stage 1; (2) target population; (3) psychometric terms; and (4) cancer terms (see “Appendix 2” for the search strategy used for MEDLINE and adapted for other databases). Searches were run on 16 January 2015 (CINAHL and PsycINFO) and 5 February 2015 (MEDLINE and EMBASE).

### Study selection criteria

Studies that reported the reliability, validity, responsiveness, precision and/or acceptability of the caregiver impact measure and met the inclusion and exclusion criteria listed above were selected for this review. Cross-cultural studies were included only if referencing an English language version of the instrument. Searches were not limited by study design or date, but were limited to articles and instrument use in the English language.

**Fig. 2:**
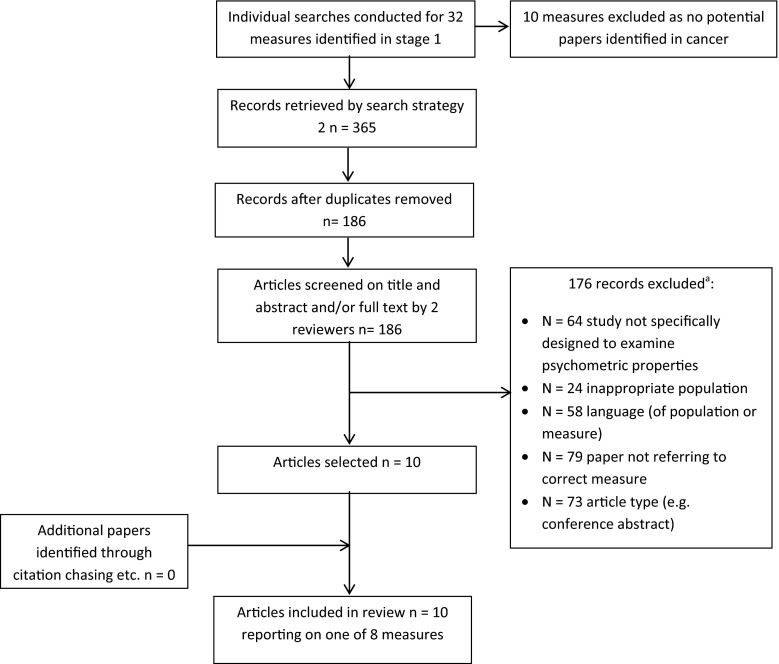
Flow chart showing study selection in search stage 2. ^a^ All breaches of exclusion criteria were recorded; articles were excluded for multiple reasons

In addition to the exclusion criteria from stage 1, papers were excluded if: the instrument was used as a “gold standard” to test other measures; psychometric evidence was reported incidentally in studies not designed to evaluate those properties; studies addressing preference weighting or scaling issues for preference-based measures; editorials, opinions, letters and meeting abstracts. Titles and abstracts were screened independently by two reviewers (VS/LM, Fig. 2).

### Citation chasing

Backwards citation chasing (one generation) using reference lists of all studies included in this stage of the review and forwards citation chasing (one generation) using Science Citation Index Expanded and Social Science Citation Index Expanded via Web of Science identified no additional eligible studies.

### Data extraction

For each included measure, we extracted: name of measure and acronym, key reference/development paper, purpose of measurement, number of items, completion time, response options, recall period, population originally developed with and types of domains/dimensions assessed.

For each included paper, the following descriptive data were extracted: instrument version, first author name, publication year, study aim, study population, number of participants and setting/country where the study was conducted. Any data on evidence of the psychometric properties or performance of instruments were extracted including content validity (theoretical framework and/or qualitative research), construct validity (structural validity and hypothesis testing), internal consistency, test–retest reliability, precision, responsiveness and acceptability. Data were extracted by one reviewer (LM/VS) and checked by a second reviewer (LM/VS).

### Evidence for psychometric performance

Evidence of psychometric performance was compared to reference criteria for (1) content validity (qualitative research with potential respondents and involvement in development stage and item generation, clear conceptual framework); (2) construct validity assessed through convergent and divergent validity demonstrated by the ability to differentiate known groups, and/or a pattern of correlation between the scale and other measures; (3) structural validity from factor analysis; (4) criterion validity (concurrent validity assessed through correlation with a gold standard and/or predictive validity where the predicted strength and direction of correlations/direction of group differences should be identified a priori); (5) reproducibility/test–retest reliability (intraclass correlation coefficient >0.7 adequate, >0.9, excellent); (6) internal consistency (Cronbach’s alpha coefficient 0.7 ≤ *α* ≥ 0.9, item total correlations >0.2); (7) responsiveness (change pre–post intervention statistically significant and/or difference of expected magnitude); (8) precision (assessment of measurement error, floor or ceiling effects <15 %; evidence from Rasch analysis); and (9) acceptability (non-response/non-completion of questionnaires, proportion of missing data) [8].

For each property, the paper was given a rating of 0 if it did not evaluate or report the property, ~ if the property was evaluated and met the criteria partially (e.g. not for all domains), + if the property was evaluated and met the criteria and – if the finding went against the prediction. Judgements on whether criteria were met were made by two reviewers (VS/LM) with disagreement resolved in discussion with another reviewer (LJF/VJ) where necessary. Content validity is only appraised for papers reporting measure development.

### Examination of instrument content and categorisation into related domains

Individual scale items from all included measures were systematically categorised by the authors into conceptual domains. Initial domains were identified from the literature, and additional domains were defined until all individual items had been mapped. The content of each was then reviewed by the team to ensure that the concepts were consistently applied and had face validity [9].

## Results

The purpose of stage 1 was to generate a list of eligible candidate instruments. Thirty-two were identified (Fig. 1), and in stage 2, individual searches were conducted for each. Ten measures were excluded as no candidate papers were returned. The combined searches for each of the remaining 22 individual measures resulted in 365 records. After deduplication, 186 unique records were screened. One hundred and seventy-six were excluded because they did not meet inclusion criteria resulting in the inclusion of 10 papers that reported on the psychometric properties of 8 eligible measures in the cancer caregiver population (see Fig. 2 for full details of reasons for exclusion). Table 1 details the general characteristics of the 8 included measures, including full name and acronym. We refer to measures by the acronym. Of the 8 included measures, 5 were initially developed for cancer caregivers, 2 of which were specifically developed for use in the palliative setting [10, 11]. Some measures were developed relatively recently [10–12], five between 1980 and 1999 [13–17]. Two [10, 13] measure caregiver appraisal specifically, with a theoretical underpinning from the stress and coping model of Lazarus and Folkman [18]; two were designed to measure subjective burden ± distress [12, 17] (the Zarit Burden Interview was later revised [19]); three were multidimensional quality of life measures [11, 14, 16] and one a multidimensional measure of caregivers’ reactions to caring for a family member [15].

**Table 1:** General characteristics of identified measures

Name and acronym	Initially developed in cancer population?	Key reference paper	Purpose of measure	Initial test population	Number of items	Completion time	Recall period	Response options	Primary area of measurement	Domains assessed (author identified)
Appraisal of caregiving scale (ACS)	Y	Oberst et al. [13]	To assess the intensity of four possible appraisals of caregiving	47 family caregivers of adults receiving radiotherapy	27 (original scale, 53)	Not stated	7 days	5-point Likert point scale 1 = strongly disagree to 5 = strongly agree	Appraisal	Threat (13 items), benefit (6 items), general stress (8 items) [original scale threat (15 items), harm/loss (15 items), challenge (15 items), benign (8 items)]
Brief Assessment scale for Caregivers of the Medically Ill (BASC)	N	Glajchen et al. [12]	To develop a brief validated instrument that can measure caregiver distress and burden in a clinical setting	Caregivers of patients with cancer (*n* = 56), neurological conditions (*n* = 16), mental health (*n* = 12) musculoskeletal (*n* = 2) COPD (*n* = 1) multiple conditions (*n* = 15)	14	Not stated	1 month	Varied: all questions had a four-point scale (not at all—a lot; not at all distressed—a lot of distress; agree a lot—disagree a lot). Ten items had an additional option of “does not apply”	Distress and subjective burden	Negative personal impact (5 items), positive personal impact (3 items), other family members (2 items), medical issues (3 items), concerns about loved ones (2 items) [identified from factor structure rather than specifically stated as domains, one item loads on two factors] Negative personal impact identified as independent subscale
Caregiver Quality of Life Index—Cancer (CQOLC)	Y	Weitzner et al. [14]	Quality of life instrument for the family caregiver of persons with cancer	263 primary family caregivers of lung, breast or prostate cancer patients	35	10 min	7 days	5-point Likert-type scale ranging from 0 = not at all to 4 = very much	Multidimensional quality of life	Burden (10 items), financial (3 items), disruptiveness (7 items), positive adaptation (7 items) [identified from factor structure rather than specifically stated as domains—not all items load]
Caregiver reaction assessment (CRA)	N	Given et al. [15]	Multidimensional instrument to assess the reactions to caring for a family member	276 caregivers to people with cancer, 101 caregivers to people with Alzheimer’s disease	24	<10 min	Asked to rate the perceived impact of caregiving but not a specified timescale	5-point Likert-type scale ranging from 1 = strongly disagree to 5 = strongly agree	Multidimensional measure of reaction to caring for a family member	Financial problems (3 items), disrupted schedule (5 items), lack of family support (5 items),health problems (4 items), self-esteem (7 items)
Family Appraisal of Caregiving Questionnaire for Palliative Care (FACQ-PC)	Y	Cooper et al. [10]	Provides a measure of the family’s appraisal of caregiving that can be used in clinical assessment	160 family caregivers of a relative with cancer receiving home-based palliative care	25	Not measured (completed in care agency where they were distributed or at home)	2 weeks	5-point Likert-type scale 1 = strongly disagree to 5 = strongly agree	Appraisal	Caregiver strain (8 items), caregiver distress (4 items), family well-being (6 items), positive care appraisals (7 items)
Quality of Life in Life-Threatening Illness—Family Carer Version (QOLLTI-F)	Y	Cohen et al. [11]	Self-report instrument to measure quality of life in carers of people receiving palliative care	245 carers of cancer patients including lung (22 %), GI (18 %), genitourinary (15 %) and other cancers (45 %)	16	Not stated	2 days (48 h)	11-point scale with a range of response options	Multidimensional quality of life	Financial (1 item), state of carer (5 items), patient well-being (1 item), quality of care (2 items), carer’s outlook (3 items), environment (2 items), relationships (2 items)
Quality of Life—Family version (formerly Quality of Life Family Caregiver Tool) (QOL-F)	Y	Ferrell et al. [16]	To provide a parallel outcome measure to compare patient and family caregiver well-being	231 family caregivers of patients with cancer pain receiving home care	37 (original scale 27)	Not stated	At this time	11-point scale with a range of response options	Multidimensional quality of life	Social concerns (9 items), physical well-being (5 items), psychological well-being (16 items), spiritual well-being (7 items)
Zarit Burden Interview (ZBI)	N	Zarit et al. [17] (revised version of scale Zarit et al. [19])	To assess the level and sources of burden experienced by caregivers to facilitate the development of interventions to reduce burden	29 primary caregivers to people with senile dementia	22 (reduced from an initial 29)	Not stated	Asked to rate the perceived impact of caregiving but not a specified timescale	5-point Likert-type scale ranging from never = 0 to nearly always = 4	Subjective burden	Questions were selected to include caregiver’s health, psychological well-being, finances, social life and the relationship between the caregiver and the patient. At least 2 factors; personal strain and role strain have been identified within the general construct of subjective burden measured by this scale

Table 2 describes the ten studies reporting on the psychometric properties of the measures in the cancer caregiver population in terms of the instrument and version, study aim, population, setting country and number of participants.

**Table 2:** Studies evaluating psychometric performance of measures in the cancer caregiver population

Measure	Version	First author	Year	Study aim	Population	*N*	Setting/country	Administration
ACS	27-item	Lambert [21]	2015	To examine the psychometric properties of the ACS	Adult caregivers of advanced breast, colorectal, lung or prostate cancer diagnosed in the last six months	484	USA	Reports baseline data from 484 participants and T2 data from 163 participants. Competed alongside other measures of caregiver burden, depression, benefit finding, coping, dyadic support and hopelessness
ACS	Original 53-item (reduced to 27 after this analysis)	Oberst [13]	1989	To examine family caregiving demands and caregivers’ appraisals of the caregiving experience	Family caregivers of adults receiving radiotherapy as outpatients	47	USA	Single time point alongside a measure of time spent in caregiving (also a new measure)
BASC	Original 43-item (reduced to 14 items after this analysis)	Glajchen [12]	2005	To develop a brief validated instrument that can measure caregiver distress and burden in a clinical setting	Caregivers of patients with cancer (*n* = 56), neurological conditions (*n* = 16), mental health (*n* = 12) musculoskeletal (*n* = 2) COPD (*n* = 1) multiple conditions (*n* = 15)	102	USA	Single time point alongside validation instruments measuring objective and subjective burden, spiritual health, physical health, satisfaction with patient care, social support, unmet need, mental health and impact of caregiving on quality of life (single item)
CQOLC	35-item	Weitzner [14]	1999a	To evaluate the reliability and validity of the caregiver quality of life index—cancer	Primary family caregivers of patients with breast, lung or prostate cancer	263	USA	180 participants completed the questionnaires once; 83 (different) participants completed the questionnaires on two occasions 14 days apart. Questionnaire packs also include measures of perceived health and functioning, depression, anxiety, burden, social support, social desirability and a single-item measure for proxy reporting of the patient’s physical status and ambulatory ability
CQOLC	35-item	Weitzner [22]	1999b	To revalidate the caregiver quality of life instrument in home hospice care and demonstrate generalisability	Family members of cancer patients who were receiving hospice services at home	239	USA	Participants completed the questionnaire at a single time point along with a measure of perceived health and functioning (SF-36), a four-item quality of life instrument using a visual analogue scale and a single-item measure for proxy reporting of the patient’s physical status and ambulatory ability
CRA	40-item (reduced to 24 in this analysis)	Given [15]	1992	Exploratory and confirmatory factor analysis^a^ of new measure, test factorial invariance and construct validity	Caregivers of patients with physical impairment (*N* = 267), Alzheimer’s disease (*N* = 211), cancer (*N* = 276)	754	USA	Most participants completed at a single time point. 193 participants completed at 3 time points, baseline, 6 months and 12 months. CRA was completed alongside a measure of depression and activities of daily living
FACQ-PC	26-item (reduced to 25 items after this analysis)	Cooper [10]	2006	To develop and validate a scale designed to measure the positive and negative components of caregiving for family caregivers of people receiving palliative care at home	Adult family caregivers of a relative receiving palliative care	160	Australia	Single time point. 160 caregivers completed the FACQ-PC, a subsample of 56 also completed validation measures of family functioning, positive and negative effect and subjective burden
QOL-F	37-item version	Sherman [23]	2006	To establish the reliability of the quality of life instrument based on patients with AIDS, patients with cancer and AIDS and cancer family caregivers. To identify differences in quality of life between patients with AIDS and cancer and their family caregivers	81 family caregivers (38 cancer, 43 AIDS) 101 patients (38 cancer, 63 AIDS)	38 (only data on cancer caregivers used in this review)	USA	Presents data from baseline and month 3 of a longitudinal study. Only the target instrument appears to have been administered
QOLLTI-F	24-item (reduced to 16 items after this analysis)^b^	Cohen [11]	2006	To develop and test measures of quality of life for family caregivers of palliative care patients	Primary caregivers of palliative care patients	245	Canada	Data collected at three time points. Measure completed alongside a two-item global measure of QoL
ZBI—multiple short versions	22-item	Higginson [20]	2010	To assess the validity of 6 short-form versions of the Zarit Burden Interview in 3 caregiver populations	Caregivers of patients with advanced cancer (*n* = 105), dementia (*n* = 131) acquired brain injury (*n* = 215)	105 (only data on cancer caregivers used)	UK	Data presented from one time point only. Short forms of ZBI compared with 22-item version as gold standard

^a^The data on confirmatory factor analysis are also reported by Stommel and colleagues [24] which was identified in forward citation searches. This paper is excluded as it concerns the same data

^b^Authors have now produced a second version of this questionnaire with minor changes and an additional question; however, the validation studies are as yet unpublished

### Psychometric performance

Appraisal of the psychometric performance reported in each paper is given in Table 3.

**Table 3:** Appraisal of measure performance and characteristics in the cancer caregiver population

Measure and version	References	Content validity^a^	Criterion validity	Structural validity	Construct validity	Test–retest reliability	Internal consistency	Responsiveness	Precision	Acceptability
ACS (27 item)	Lambert et al. [21]		~	+	~	0	+	0	0	0
ACS (original 53 item)	Oberst et al. [13]	~	0	0	0	N/A	~	N/A	0	+
BASC	Glajchen et al. [12]	+	0	~	~	N/A	+	N/A	0	0
CQOLC	Weitzner et al. [14]	+	0	0	+	+	+	~	0	~
CQOLC	Weitzner and McMillan [22]		0	0	+	N/A	+	–	0	~
CRA	Given et al. [15]	+	0	+	+	0	+	0	0	~
FACQ-PC	Cooper et al. [10]	~	0	+	~	N/A	+	N/A	0	0
QOL-F^b^ (37 item)	Sherman et al. [23]		0	0	0	0	+	0	0	0
QOLLTI-F	Cohen et al. [11]	+	+	+	0	~	~	~	~	~
ZBI—multiple short versions^b^	Higginson et al. [20]		+	0	+	N/A	+	N/A	0	0

0 = not evaluated, ~ = partially met criteria, + = met criteria, − = finding went against prediction

^a^Content validity is only appraised for papers reporting measure development

^b^Cancer caregiver group only with exception of discriminative validity which was calculated for all participant groups in ZBI study

#### Content validity

Of the six studies describing measure development, content validity was generally well described and acceptable. Four (BASC, CQOLC, CRA, QOLLTI-F) describe qualitative work with potential respondents for item development and reduction [11, 12, 14, 15]; two (ACS, FACQ-PC) describe a clear underpinning conceptual framework but no involvement of potential respondents [10, 13].

#### Criterion validity

Concurrent validity: the 6 short forms of the ZBI were validated against the 22-item version as gold standard. Spearman rank order correlations ranged from 0.63 for the one-item version to 0.95 for the 12-item scale [20]. Concurrent validity of the ACS Benefit subscale only was assessed against the Benefit Finding Scale as the gold standard (*r* = 0.56) [21].

Predictive validity: predictive validity of the ACS was assessed against hopelessness and depression scores at time 2 [21]. Although overall a significant amount of variance in hopelessness (33.3 %) and depression (27.8 %) was explained by ACS scores at time 1, only half of the predictive validity hypotheses were supported. Criterion validity of the QOLLTI-F was assessed using a 2-item measure of global quality of life. QOLLTI-F was predicted between 43 and 55 % of the variance depending on whether individual items (55 %), subscale scores (53 %) or total score (43 %) was regressed.

#### Structural validity

Structural validity using factor analysis was described in five of the studies. For the CRA [15], exploratory factor analysis supported the five-subscale solution accounting for 65.1 % of variance. Confirmatory factor analysis demonstrated factorial invariance across disease (cancer vs dementia), caregiver type (spouse vs non-spouse) and over time. For the QOLLTI-F [11], the authors describe an acceptable seven-factor solution with exploratory factor analysis (although the total amount of variance explained is not reported) with factor loadings from 0.39 to 0.88. For the FACQ-PC [10], principal axis factor analysis supports a four-factor solution with factor loadings ranging from 0.33 to 0.92. Although all items load highest on the predicted factor, two items cross load (>0.3). Lambert and colleagues [21] report a three-factor solution for the ACS which supports the original subscales, had minimal cross-loadings and factor loadings ranging from 0.405 to 0.726. Glajchen et al. [12] report a five-factor solution for the BASC while noting that one item cross loads. The authors do not report their methods or the factor loadings.

#### Construct validity: hypothesis testing

Six studies assessed construct validity through convergent and divergent validity. For the ACS, only 5/12 correlations between subscales of the ACS and other measures exceeded the authors’ criterion of ±0.3 to demonstrate construct validity [21]. Both papers assessing the CQOLC report moderate-to-high correlations with measures completed at the same time [14, 22]. Only the initial validation study assessed divergent validity using dissimilar measures and found that these gave low correlations with CQOLC scores as expected [14]. Construct validity of the BASC was supported by an appropriate pattern of moderate–strong correlations with similar measures [12].

Strong correlations were found between subscales of the FACQ-PC [10] and measures used to test convergent validity; however, positive caregiving appraisals were only weak–moderately associated with positive affect (*r* = 0.3). Similarly, to demonstrate divergent validity, four correlations were calculated between subscales and other measures which should yield low, negative correlations. While all were negative, two correlations were moderate in magnitude (*r* = −0.4 and *r* = −0.38).

Construct validity for the CRA [15] was assessed by correlating subscale scores with caregiver depression and patient dependencies in activities of daily living (ADL). The five subscales were, as predicted, weakly correlated with patient dependencies in ADL. Correlations with depression were in the appropriate direction and ranged from −0.23 to 0.57 in magnitude.

Three studies conducted hypothesis testing by assessing “known-group” differences. Group differences analysis for the ACS was only partially supportive of construct validity with only 3/9 hypotheses significant [21]. The BASC was able to discriminate between male and female caregivers and between different relationships between caregivers and patients. The negative personal impact subscale, but not the total score, differentiated between caregivers with and without mental health conditions. There were weak correlations overall with depression, high blood pressure and gastrointestinal complaints. All short forms of the ZBI were shown to have good discriminative validity to correctly classify participants as those with and without burden (contrasting to the classification on the 22-item version as gold standard).

Two studies [13, 23] report comparisons between groups (e.g. male/female; spouse/non-spouse), but these were not established a priori as known-group differences for hypothesis testing. One did not examine construct validity with convergent, divergent or known-group analysis [11].

#### Internal consistency

Internal consistency was assessed in all papers. All subscales of the ACS had *α* > 0.7 in both papers [13, 21] with the exception of the challenge subscale, which was subsequently dropped from the measure [13]; the threat subscale slightly exceeded the upper limit of *α* at 0.91 in one paper. [13] Overall *α* for the BASC was just acceptable (0.7); the negative personal impact factor, which can be used as an independent subscale, was 0.8. For the CQOLC, *α* approaches and slightly exceeds the upper limit (*α* = 0.87 and 0.91, respectively) [14, 22]. The five subscales of the CRA range from *α* = 0.8 to 0.9 [15] and the four subscales of the FACQ-PC from *α* = 0.73 to 0.86. Item total correlations were all in excess of 0.2, the strongest 0.78 [10]. Overall *α* for the QOLLTI-F was 0.86. The individual subscales were generally weaker ranging from *α* = 0.48 to 0.81 which may reflect the small number of items in some subscales. The measure also includes two single-item subscales [11]. Internal consistency for the QOL-FV was *α* = 0.89. Finally, internal consistency for the ZBI 22-item version as gold standard was *α* = 0.88 and ranged from 0.69 for the 4-item short version to 0.85 for the 12-item version.

#### Test–retest reliability

Three of the five papers with at least two time points did not attempt to assess test–retest reliability [15, 21, 23]. Test–retest reliability of the CQOLC was found to be excellent (0.95) [14]. For the total QOLLTI-F, test–retest reliability was found to be acceptable between T1 and T2 (0.77) and T2 and T3 (0.80). Intraclass correlations for individual subscales were below an acceptable level in 10 out of 14 cases, which may reflect the small number of items in the subscales [11].

#### Responsiveness

Responsiveness of the QOLLTI-F [11] was assessed by contrasting subscale scores on days that participants considered to be bad, average and good. These differences were statistically significant in all comparisons with the exception of the financial concerns subscale between average and good days. All differences between good and bad days exceeded 0.5 s.d. for minimal important difference. Only 3/8 comparisons did so between good and average and 4/8 between average and bad days.

The “potential to be responsive to change” of the CQOLC was assessed by using CQOLC to predict patient performance status at a single time point rather than measure responsiveness to change over time. The studies report contradictory findings: in one [14], the predicted significant negative correlation between CQOLC scores and patient performance status is reported as significant (*r* = −0.46, *p* < 0.0001), but in the other [22], this correlation approaches zero (*r* = 0.09).

#### Precision

None of the included studies conducted Rasch analysis or an assessment of measurement error. Floor and ceiling effects were not formally reported in any paper although two subscales of the QOLLTI-F [11] were described as having a lack of variance due to ceiling effects which made them less predictive of global quality of life. The subscales, quality of care and relationships, comprised two items each.

#### Acceptability

The acceptability of measures was not consistently reported and was difficult to assess using missing data and participation rates, as the measure is often given as part of a pack and information is not assessed separately. No information pertaining to acceptability was provided by four studies [10, 20, 21, 23]. In five studies, acceptability was appraised as only partially evidenced due to high dropout or incomplete data [11, 12, 14, 15, 22], surprising for the QOLLTI-F which had thoroughly tested acceptability in the development phase [11]. For the ACS [13], overall response rate was 74 % (including postal responses) and only 3/50 participants were eliminated due to missing data, suggesting the questionnaire was acceptable.

### Examination of instrument content and categorisation into related domains

The 8 included instruments yielded 194 individual items. These were categorised into 16 conceptual domains under 5 overarching themes of approximately equal size: lifestyle disruption (22 % of items); well-being (22 %); health of the caregiver (21 %); managing the situation (18 %) and relationships (18 %). Most dominant domains were “confidence, self-esteem and self-efficacy” (24 items across 7 measures) and “psychological health of the caregiver” (22 items across 6 measures). Least represented were “impact on other family members” (2 items across 2 measures) and “impact on paid employment” (2 items across 2 measures). The distribution and total number of items across the different domains along with example items are given in Table 4.

**Table 4:** Domains assessed by each measure

Overarching theme	Specific domain	ACS	BASC	CQOLC	CRA	FACQ-PC	QOL-F	QOLLTI-F	ZBI^a^	*N* of measures (total *N* of items)	Example items (measure and subscale/factor name where relevant)
Health of caregiver	Psychological health of caregiver	4	1	6		2	7	2		6 (22)	Over the past 2 days (48 h) emotionally I felt…(QOLLTI-F *carer’s own state*) How much have you been depressed about X’s illness? (BASC)
	Physical health of caregiver	2		1	4	2	5	1	1	7 (16)	As a caregiver I feel my own health has suffered (FACQ-PC—*caregiver strain*) I am healthy enough to care for X (CRA—*health problems*)
	Sexual activity			1			1			2 (2)	I am satisfied with my sex life (CQOLC)
Lifestyle disruption	Lifestyle disruption	3	1	5	3		1		1	6 (14)	Do you feel stressed between caring for your relative and trying to meet other responsibilities for your family or work? (ZBI) I have eliminated things from my schedule since caring for X (CRA—*disrupted schedule*)
	Impact on paid employment					1	1			2 (2)	To what degree has your family member’s illness or treatment interfered with your employment? (QOL-F—*social concerns*)
	Financial implications	1		3	3	1	1	1		6 (10)	How much financial burden resulted from your family member’s illness or treatment?(QOL-F—*social concerns*)
	Time for self, social life and leisure	2	1	1	2	2	2	2	5	8 (17)	I have had to give up my social life to care for X (FACQ-PC—*caregiver strain*) Do you feel that because of the time you spend with your relative that you don’t have enough time for yourself? (ZBI)
Relationships	Impact on relationship with care recipient	1	2	1		1		1	2	6 (8)	Caring for X has made me feel closer to him/her (FACQ-PC—*positive caregiving appraisal*) Distress of seeing how much X’s illness has changed your relationship (BASC)
	Relationships with other family members and friends:										
	Communication			1		4		1		3 (6)	Our family is able to talk about our feelings with each other (FACQ-PC—*family well-being*)
	Relationships	3	2			1	1		1	5 (8)	My relationships with friends and family are not affected by this situation (ACS—*general stress*)
	Support			3	5	1	1			4 (10)	It is very difficult to get help from my family in taking care of X (CRA—*lack of family support*)
	Impact on other family members			1			1			2 (2)	I worry about the impact my loved one’s illness has had on my children or other family members (CQOLC—*burden*)
Well-being	Confidence, self-esteem and self-efficacy	4	1		3	7	3	2	4	7 (24)	Taking care of X makes me feel good about myself (BASC) Do you feel you could do a better job in caring for your relative? (ZBI)
	Spirituality^b^			1			3	1		3 (5)	Over the past 2 days (48 h) I was comforted by my outlook on life, faith or spirituality (QOLLTI-F—*carer’s outlook*)
	Bringing purpose and meaning to life	2	1	2	3	2	2	1		7 (13)	I am glad my focus is on getting my loved one well (CQOLC—*positive adaptation*) Taking care of X has brought meaning to my life (BASC)
Managing the situation	Coping	1	1	1		1	1			5 (5)	The responsibility I have for my loved one’s care at home is overwhelming (CQOLC—*disruptiveness*)
	Concerns for the future	4		1			1			3 (6)	How much uncertainty do you feel about your family member’s future? (QOL-F—*spiritual well-being*) I worry that in the future I will not be able to help the person needing my care (ACS—*threat*)
	Concerns/distress about the health/care of recipient		3	6			6	4		4 (19)	I fear the adverse effects of treatment on my loved one (CQOLC—*burden*) Over the past 2 days (48 h) the quality of health care we received was….(QOLTTI-F—*quality of care*)
	Burden		1	1	1				2	4 (5)	Overall how burdened do you feel caring for your relative? (ZBI)
	Total number of items	27	14	35	24	25	37	16	16	194	

^a^We have only included the items from the Zarit Burden Inventory which are used in one of the short forms of the scale evaluated in the paper by Higginson et al. [20]

^b^Items categorised under the domain “spirituality” make direct reference to spirituality, faith, prayer, church/temple, etc

## Discussion

This systematic review was conducted to investigate instruments commonly used to measure caregiver impact in cancer. Specifically, we sought to identify (1) what caregivers were being asked about, and (2) whether the measures performed well in psychometric evaluation. Psychometric appraisal is critical to establish the quality and standards of a measure in a given context. With so many instruments available to researchers, this review is intended as a resource to enable researchers to judge for themselves whether the content and quality of the instruments described match their requirements.

For 24 of the 32 identified measures, we found no evidence of psychometric performance using English language versions with cancer caregivers (see electronic supplementary material for a list of these measures). This is not to say that the measures have not been evaluated, but that we found no evidence in cancer. When assessing the performance of an instrument, the context is critical as it may perform differently in other populations. Without evaluation in cancer, researchers cannot be sure that instruments are reliably measuring the intended constructs. For 6 of the remaining 8 questionnaires, evidence of psychometric performance was identified in only a single study. The small number of studies identified meant that the evidence for psychometric performance was appraised for each individual paper, rather than allowing the appraisal to consider performance across a group of studies. In particular, the lack of studies beyond the initial validation of some measures is of concern. Most studies collected data at a single time point, and so, responsiveness to change, test–retest reliability and measurement error were not assessed. For the most part, content validity and internal consistency were reported and were adequate. Structural validity was assessed using factor analysis in five studies; four met these criteria, one only partially. Construct validity was assessed using different approaches to hypothesis testing in seven papers, fully meeting the criteria in only three. In the limited number of papers included, strongest support for psychometric performance was reported for the CRA and CQOLC.

We set out to examine not only the psychometric performance of these measures in a cancer population, but also to understand what concepts and domains were being assessed. Only one of the 16 conceptual domains, *time for self, social life and leisure*, was represented in all eight instruments. There was considerable overlap in the domains measured, however, with 8/16 domains being assessed in at least 6/8 measures. We have identified several areas which are not well captured by the instruments included in this review.

### Paid employment

Impact on paid employment was assessed with a single question on two measures, neither of which addressed impact on career aspiration and planning or career progression, simply whether paid employment had been affected (FACQ-PC [10], QOL-F [16]).

### Sexual activity

Only two questionnaires ask about relationships in terms of sexual activity (CQOLC [14], QOL-F [16]).

### Family members

We also found that impact on the family as a unit was not well covered in the current measures. Impact on other family members was only addressed with a single question on two questionnaires (again CQOLC [14] and QOL-F [16]).

Current scales do not adequately capture role changes and responsibilities in the household and family routines, for example the impact on other caregiving responsibility such as to children or parents. Taking on new roles and responsibilities can raise issues around feelings of competence in the role, role strain and conflict and family cohesion. Such changes may be fluid as the cared-for member of the family moves through different phases of their cancer and treatment.

Five of the eight measures included in this review were developed between 1980 and 1999. There has been considerable societal change in the intervening years; families, roles and responsibilities are structured differently. It is not clear how appropriate some of the older questionnaires are for the present day. The activities associated with informal caregiving incorporate a range of tasks affecting different aspects of the life of the caregiver and the whole family [25, 26]. The impact of caregiving will vary depending not only on the patient’s situation but also on family make-up, in terms of other caregiving responsibilities, financial and occupation role responsibilities and time of life [6, 27, 28]. Impact is also likely affected by the *number* of other social roles, such as employment and other caregiving responsibilities that the caregiver has [3]. There is limited research about how the effect on variables such as employment and role strain might change over time, as caring responsibilities likely vary in line with different lines of treatment or transition to palliative care [27, 29, 30]. Future measures should attempt to capture the changing nature of caregiver impact.

We have identified a number of areas which are currently not well captured by measures that have been evaluated in cancer. These gaps may exist for several reasons. First, some measures were not initially developed for this population, and so, constructs important to cancer caregivers may not have the same salience. Second, some measures are old and may not reflect what is important in current society. Third, advances in cancer treatments mean that many more people are living a long life with cancer. For some, initial therapy is just the start of a journey that will involve repeated lines of treatment over time. The patient and the whole family have to continually adjust to a fluid situation and will be impacted variably at different times while trying to maintain a sense of normality in other aspects of their lives. Better treatments mean a longer life with cancer is a possibility for patients; however, we need ways to measure the longer-term impacts of cancer and cancer treatment for them and their informal caregivers. At the very least, we would suggest caregiver input into updating some of the older content if not the development of a new measure to capture the broader impacts we have described.

### Limitations

The search strategy may have limited the number of papers identified in two ways: (1) searching for measures by name and acronym. The precise wording of the measure name and even the acronym sometimes varied; (2) reporting standards have changed; some older papers have poor use of keywords and do not always include psychometric terms or the names of measures in title/abstract/keywords. The impact of both of these limitations is mitigated by thorough backwards and forwards citation chasing.

We intentionally restricted the review to studies that reported on the psychometric properties of the English version of measures. This decision was taken as we felt we could not assume cultural equivalence for the caregiving role or the salience of different aspects of burden and impact in diverse populations. We took the decision to exclude all non-English versions of the measures rather than make subjective decisions as to whether one culture was sufficiently similar, while another was not. We are aware, however, that there are a number of studies reporting on the psychometric properties of other language versions of measures included in this review, e.g. [31–38]. We acknowledge there may be cultural differences between and within different countries where English is commonly spoken and where measures developed in English have been used. While this is an extremely important area of research, it is beyond the remit of the current review and it is not an aim of this study to investigate these potential differences. In this review, 7/10 included studies were conducted in the USA and 1 study each in the UK, Australia and Canada.

We also recognise that the pool of individual items identified is restricted by our stringent inclusion criteria for measures. Measures developed in other contexts, e.g. family function in a paediatric setting [39], for economic evaluation [40], domain-specific measures [41] and multidimensional measures which have not been subject to psychometric evaluation in cancer caregivers in the English language [6, 42, 43] may include concepts and items that are pertinent but which would need to be evaluated in appropriate studies.

## Conclusions

A large number of measures purport to assess caregiver impact, but most have not been subject to psychometric evaluation in cancer populations. Few studies met our inclusion criteria so it was not possible to consider psychometric performance of the measures across a group of studies. Our content analysis identified several areas which are currently not well captured. These include changes to career aspiration and planning, changes in roles and responsibilities within the family and the way the family functions as a unit. We also note that some of the measures were developed up to 35 years ago, and their relevance to the current day may need to be reviewed. Strategies to overcome some of these limitations could include caregiver input into revising existing measures or using two or more measures to cover a broader range of outcome domains. However, our review suggests there is a need for a new measure capturing the impacts on broader areas of life for the caregiver and the family unit.

### Electronic supplementary material

Below is the link to the electronic supplementary material.

Supplementary material 1 (DOCX 14 kb)

## Appendix 1: Search strategy phase 1 (used in MEDLINE and adjusted for other databases)

caregivers MedLine final 20th Nov

1. exp “outcome assessment (Health Care)”/
2. tool.ti,ab.
3. instrument.ti,ab.
4. questionnaire.ti,ab.
5. index.ti,ab.
6. indices.ti,ab.
7. scale.ti,ab.
8. survey.ti,ab.
9. interview.ti,ab.
10. inventory.ti,ab.
11. outcome assessment.ti,ab.
12. outcome measure.ti,ab.
13. (measur* adj4 (quality or health or impact or burden or well being or wellbeing or lifestyle or family function or experience)).ti,ab.
14. (assess* adj4 (quality or health or impact or burden or well being or wellbeing or lifestyle or family function or experience)).ti,ab.
15. or/1-14
16. exp “quality of life”/
17. quality of life.ti,ab.
18. health outcome*.ti,ab.
19. health status.ti,ab.
20. (well being or wellbeing).ti,ab.
21. ((caring or caregiving or caregiver* or carer*) adj2 impact).ti,ab.
22. ((caring or caregiving or caregiver* or carer*) adj2 burden).ti,ab.
23. ((caring or caregiving or caregiver* or carer*) adj2 experience).ti,ab.
24. ((caring or caregiving or caregiver* or carer*) adj2 stress).ti,ab.
25. ((caring or caregiving or caregiver* or carer*) adj2 strain).ti,ab.
26. health utility.ti,ab.
27. lifestyle interference.ti,ab.
28. family function*.ti,ab.
29. or/16-28
30. exp “caregivers”/
31. (carer or caregiver).ti,ab.
32. ((family or spouse or husband or wife or partner or friend) adj5 caring).ti,ab.
33. ((child* or son or daughter or parent or relative or relation) adj5 caring).ti,ab.
34. or/30-33
35. exp “reproducibility of results”/
36. exp “psychometrics”/
37. reliab*.ti,ab.
38. valid*.ti,ab.
39. psychometric.ti,ab.
40. or/35-39
41. 15 and 29 and 34 and 40
42. limit 41 to english language

## Appendix 2: Search Strategy to identify evidence of psychometric performance of candidate instruments when used with caregivers to cancer patients (used in MEDLINE and adjusted for other databases)

- Database: Ovid MEDLINE(R) In-Process & Other Non-Indexed Citations and Ovid MEDLINE(R) <1946 to Present>
- Search strategy:
  1. caregivers/
  2. (carer* or caregiver*).ti,ab.
  3. ((family or spouse or husband or wife or partner or friend) adj5 caring).ti,ab.
  4. ((child* or son or daughter or parent or relative or relation) adj5 caring).ti,ab.
  5. or/1-4
  6. reliab*.ti,ab,kw.
  7. valid*.ti,ab,kw.
  8. evaluat*.ti,ab,kw.
  9. repeatab*.ti,ab,kw.
  10. acceptab*.ti,ab,kw.
  11. responsiv*.ti,ab,kw.
  12. feasib*.ti,ab,kw.
  13. psychometr*.ti,ab,kw.
  14. or/6-13
  15. neoplasm/
  16. cancer.ti,ab.
  17. oncology.ti,ab.
  18. 15 or 16 or 17
  19. 5 and 14 and 18
- For each candidate instrument: 19 AND [Name of measure, including variants & acronyms]
