# Supplementary material for: Patient-reported outcome measures for cancer caregivers: a systematic review
Source: Qual Life Res. 2016 Feb 12;25:1859–76. doi: 10.1007/s11136-016-1239-0 (PMC4945691; doi:10.1007/s11136-016-1239-0)
Supplement: Supplementary file 1 — Supplementary material 1 (DOCX 14 kb) [file 11136_2016_1239_MOESM1_ESM.docx]

Supplementary information

List of 24 measures for which no eligible papers were identified at stage 2 searches.

| Adult Carers Quality of Life Questionnaire (AC-QoL) |
| --- |
| Bakas Caregiving Outcome Scale (BCOS) |
| Burden Assessment Scale |
| Burden Scale for Family Caregivers (BSFC-short/BSFC-s) |
| Care Work Impact Appraisal questionnaire |
| Caregiver Burden Inventory |
| CareGiver Oncology Quality of Life questionnaire (CarGOQoL) |
| Caregiver Quality of Life Index (CQLI) |
| Caregiver Risk Screen |
| Caregiver Strain Index (CSI) |
| Caregiver Wellbeing Scale (CWBS) |
| Caregiving Appraisal Scale |
| Caregiving Impact Scale |
| Carer Experience Scale |
| CarerQoL |
| Carers Assessment of Difficulties Index (CADI) |
| Family Reported Outcome Measure (FROM-16) |
| The Financial Impact Scale |
| Montgomery Borgatta Caregiver Burden Scale |
| Perceived Caregiver Burden scale (PCB-31) |
| Picot Caregiver Rewards Scale (PCRS) |
| Positive Aspects of Caregiving (PAC) |
| Psychological Well-being Scale for Caregivers (PWS-C) |
| Work Productivity and Activity Impairment Questionnaire |
